# Analysis methods for meso- and macroporous silicon etching baths

**DOI:** 10.1186/1556-276X-7-398

**Published:** 2012-07-17

**Authors:** Julia B Nehmann, Sarah Kajari-Schröder, Detlef W Bahnemann

**Affiliations:** 1 Institute for Solar Energy Research Hamelin (ISFH), Am Ohrberg 1, Emmerthal, 31860, Germany; 2 Institut für Technische Chemie, Leibniz Universität Hannover, Callinstrasse 3, Hannover, 30167, Germany

**Keywords:** chemical analysis, silicon etching, bath composition, organic content, hydrofluoric acid., 88.40.H solar cells (photovoltaics), 88.40.jj silicon solar cells, 82.45.Gj electrolytes.

## Abstract

Analysis methods for electrochemical etching baths consisting of various concentrations of hydrofluoric acid (HF) and an additional organic surface wetting agent are presented. These electrolytes are used for the formation of meso- and macroporous silicon. Monitoring the etching bath composition requires at least one method each for the determination of the HF concentration and the organic content of the bath. However, it is a precondition that the analysis equipment withstands the aggressive HF. Titration and a fluoride ion-selective electrode are used for the determination of the HF and a cuvette test method for the analysis of the organic content, respectively. The most suitable analysis method is identified depending on the components in the electrolyte with the focus on capability of resistance against the aggressive HF.

## Background

Porous silicon (PSi) is a promising candidate for the production of thin silicon solar cells in photovoltaic industries. The formation of a mesoporous double layer before epitaxial deposition of the absorber offers the possibility of cost reduction if the reuse of the substrate wafer is performed repeatedly. Another approach is the formation of macroporous silicon, which is used as an absorber for thin silicon solar cells and, therefore, does not require an additional epitaxial grown silicon layer [1]. Both processes have the porous layers that are etched electrochemically in hydrofluoric acid (HF) containing electrolytes in common.

Recently, there has been an increased interest in processes for thin silicon solar cells. One of these is the PSi process, which has been initially presented decades ago [2-4]. An essential part in this process is the etching of the porous silicon double layer, which consists of a starting and a separation layer. The separation layer is the place where the deposited epitaxial layer will be detached from the substrate. The detachability is a function of the homogeneity of the porous silicon in the separation layer. Therefore, the formation of the porous layers is one of the crucial steps in the PSi process. Homogeneous pore formation can be achieved only with constant etching parameters. This can be realized by keeping the composition of the etching bath constant, as variations in the chemical composition of the bath require a modification of the etching parameters. This adjustment is a time-consuming process and requires comprehensive knowledge of the behavior of the etching process itself. By keeping the chemical composition of the bath constant, no adjustment of the etching parameters is necessary, and thus, homogeneous pore formation can be achieved. However, the chemical composition can change due to etching reactions, evaporation, dilution, or a combination of these factors. Therefore, periodical analysis of the etching bath components is required.

The chemical composition of two etching baths that are used for the formation of meso- or macroporous silicon for photovoltaic applications has been investigated. For mesoporous silicon, a highly concentrated HF solution (19.5 mol L^−1^) was used, containing ethanol as a surface wetting agent [5]. Macroporous silicon was prepared with a low-concentrated HF solution (1.5 mol L^−1^) in the presence of the surface wetting agent acetic acid [6]. Both etching processes are driven electrochemically and, therefore, do not need an additional oxidizing agent.

HF is known to be very aggressive, not only to human tissue but also to various kinds of materials, e.g., glassware or metals, which a great number of analytical instruments - at least partly - contain. This is why many established analysis methods are unsuitable for the determination of the composition of the etching baths described here, especially for the organic contents. In this paper, we will demonstrate which methods are capable of determining the HF content in various concentrations as well as how to analyze the content of the organic wetting agents, i.e., ethanol and acetic acid.

## Methods

Two methods have been established recently for HF determination in HF/HNO_3_ etching solutions [7], i.e., titration with lanthanum nitrate and detection by fluoride ion-selective electrode (F-ISE), respectively. In the following, we will show how these methods can be adapted for electrochemical etching baths of different HF concentrations, which additionally contain organic wetting agents. The organic content of the baths is determined by the cuvette test method for total organic carbon (TOC) by Hach Lange GmbH (Düsseldorf, Germany) [8].

### Analysis of the HF content

The concentration of a given aqueous sample can be determined by titration, in which a standard solution is added stepwise to the sample. Chemical reactions between the sample and the standard solution lead to a shift of the pH, which is monitored during the analysis. Therefore, titration is an indirect analysis method.

We use an InLab® Hydrofluoric electrode for pH measurements, connected to the Titrator DL28 (both from Mettler-Toledo International, Inc., Giessen, Germany). For sample preparation, the etching bath sample is diluted before analysis due to the high-HF concentration. Without dilution, the high-HF concentration would lead to a damage of the electrode and an inappropriate increase of the required volume of the standard solution.

The titration of HF is performed in two steps. First, the pH of the etching bath sample is adjusted to pH 6.5 to 7.5 with sodium hydroxide solution (1.0 mol L^−1^). This is a precondition for the second step and sets free all the fluoride ions in the sample that are bound to silicon as hexafluorosilicic acid (H_2_SiF_6_). In the second step, a standard solution of lanthanum nitrate (0.1 mol L^−1^, confirmed by measurement with a nitrate ion-selective electrode) is added stepwise, forming LaF_3_ with the freed fluoride ions. During the reaction, the pH is monitored, and the equivalence point (inflection point of pH curve) is determined. At this point, the total amount of fluoride ions in the sample is consumed in the reaction, marking the endpoint of the measurement. The required volume of lanthanum nitrate yields the amount of total fluoride in the sample. A relative error of 3.3 % for the HF determination with this titration method has been determined. The calculation of remaining free HF in the bath is possible following Equation 1:

(1) $${\left[\text{HF}\right]}_{\text{free}}={\left[\text{HF}\right]}_{\text{total}}-{\left[\text{HF}\right]}_{{\text{H}}_{2}{\text{SiF}}_{6}}$$

Equation 1 shows the amount of HF bound as H_2_SiF_6_ in the bath. The concentration of H_2_SiF_6_ can be determined with a titration method for two equivalence points. In this, a standard solution of sodium hydroxide is added, and the concentration of H_2_SiF_6_ is calculated from the volume of sodium hydroxide between both equivalence points [9,10].

Another method for the determination of HF is the F-ISE. Here, a perfectION^TM^ combination fluoride electrode from Mettler-Toledo - connected to the Titrator DL28 - is used. This electrode is sensitive for fluoride ions only, and therefore, F-ISE measurements are a direct analysis method. F-ISE is sensitive especially for low HF concentrations in the range of 0.05 to 50 mmol L^−1^ and has been calibrated according to this range. As our samples originally contain concentrations above this range, dilution is required before measurement.

For sample preparation, a buffer solution (TISAB II from Sigma-Aldrich Chemie GmbH, Steinheim, Germany) is added to the diluted etching bath sample. The adjustment of the pH to a suitable range before analysis is necessary for precise measurements. The optimal pH for F-ISE measurements is about 5.5, which is due to the formation of HF and HF_2_- and the interference from OH^-^ outside of this range [11]. We found a volume ratio of 50:1 (diluted sample:buffer) to be sufficient for pH adjustment. A relative error of 1.5 % with respect to the undiluted samples has been determined.

The accuracy of both HF determining methods described here has been verified by measurement of artificial samples with known concentrations in the respective measurement range. Furthermore, reference measurements at BASF SE (Ludwigshafen, Germany) were performed with ion chromatography and confirmed the precision of both methods within the relative error.

### Analysis of the organic content

**Figure 1:**
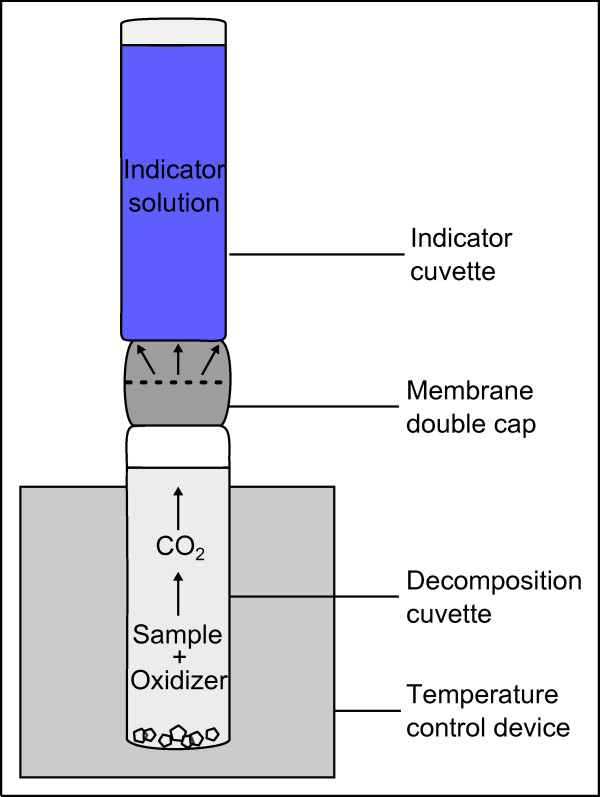
**Principle of the cuvette test method.** In order to set CO_2_ free, the sample is heated at 100 °C for 2 h [8].

**Figure 2:**
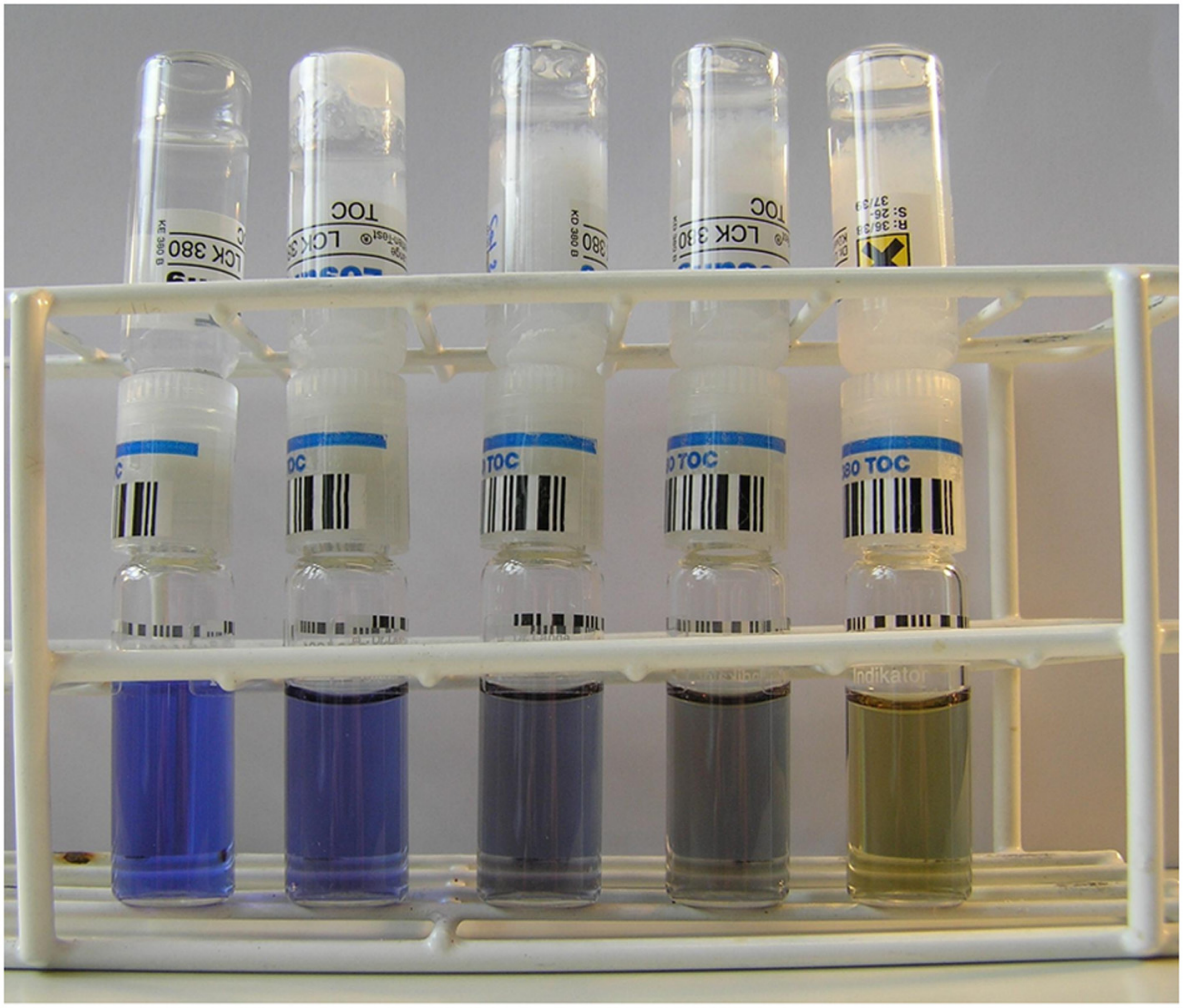
**Color change of indicator cuvettes due to the amount of TOC in the sample.** Cuvettes show zero, 10, 25, 35, 50 ppm TOC, respectively (from left to right).

The concentrations of the wetting agents are determined with a cuvette test method. Figure 1 schematically shows a typical cuvette. The lower part is a decomposition cuvette containing sodium persulfate as an oxidizing agent. The upper cuvette is filled with an indicator solution of thymol blue. We follow the difference method of Hach Lange [8]: The etching bath sample is diluted in order to fit to the measurement range and added to the decomposition cuvette. After attachment of the membrane double cap to the cuvettes, the whole cuvette system is heated up to 100 °C for 2 h. Meanwhile, the reaction proceeds between the organic compound in the sample and the oxidizing agent, and leads to the formation of CO_2_. The CO_2_ then passes the membrane double cap, which is gas permeable, leading to a color change of the indicator solution of the upper cuvette. Figure 2 depicts the different colors of the indicator solution due to the amount of CO_2_ in the system. The indicator cuvettes are evaluated by photometry (ISiS 6000 from Dr Lange) at a wavelength of 430 nm after cooling down to room temperature. The results give the amount of carbon in the sample.

The cuvette test method is sensitive for concentrations of organic compounds between 0.08 and 2.7 mmol L^−1^ and has been calibrated according to this range. The accuracy of the method has been confirmed by measurement of artificial samples.

## Results and discussion

### HF content

A comparison of parameters for titration and F-ISE is listed in Table 1. The determination of HF with titration is a convenient method for on-time analysis in the laboratory. The pH electrode requires calibration only once a week - independent on the number of samples - and each sample preparation takes only 1 min. Advantageous is the method's suitability even for HF concentrations of up to 20 mol L^−1^. Adjustment of the sample volume with respect to the HF concentration in the sample leads to results with a relative error of 3.3 %.

**Table 1:** Comparison of the parameters of the used analysis methods

**Parameter**	**Titration**	**F-ISE**	**Cuvette test**
Analyte	HF	HF	Organic content
Measurement method	Indirect	Direct	Direct
Measurement range	>50 mmol L^−1^	0.05 to 50 mmol L^−1^	0.08 to 2.7 mmol L^−1^
Time for measurement	15 min	5 min	4 h
Time for calibration	5 min^a^	15 min^b^	Only once
Suitable for acetic acid containing electrolytes?	No	Yes	Yes
Relative error	3.3 %	1.5 %	2.1 %
Standard deviation	0.1 mol L^−1^*(n = 10)*	0.2 mol L^−1^*(n = 10)*	0.01 mol L^−1^*(n = 20)*
Inline analysis possible?	Yes	Yes	No

^a^Once a week. ^b^Directly before measurement.

A disadvantage of the HF analysis by titration is the high cost of the required lanthanum nitrate. A cheaper, however, more time intensive method is the determination by F-ISE. According to Table 1, this method needs calibration directly before the measurement. Together with the longer preparation time for a sample, we find F-ISE to be less convenient for on-time analysis, although it shows a lower relative error of only 1.5 %. We find no interference of these methods regarding the organic wetting agents of the investigated etching baths.

Both methods show no interference concerning the high HF concentration, because the samples are diluted in order to pass the respective measurement ranges of the methods. Furthermore, both are capable of inline analysis in industrial production.

**Figure 3:**
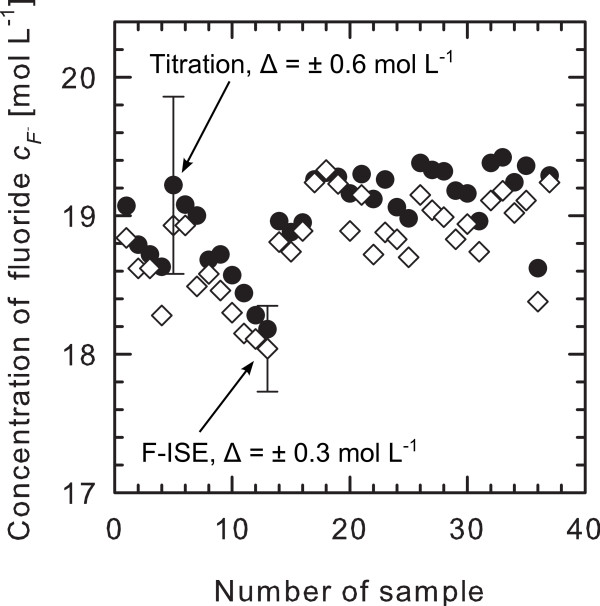
**Comparison between titration and F-ISE data points of a mesoporous silicon etching bath.** Both methods yield the same result within the measurement accuracy. The steps in HF concentration are due to replenishment of the bath.

Figure 3 shows a comparison of the number of samples measured both with F-ISE and titration. The measured samples emanate from an etching bath for mesoporous silicon, containing HF in high concentration as well as ethanol as a surface wetting agent. Both, titration and F-ISE, yield precise results with a deviation between both methods of about 0.2 mol L^−1^. Therefore, the results are consistent within the measurement accuracy. It should be noted that the lanthanum nitrate concentration was controlled with respect to the nominal concentration and the results used for the comparison of both methods. In conclusion, the HF content of the mesoporous silicon etching bath can be determined by titration as well as by F-ISE.

The steps in the HF concentration in Figure 3 are due to replenishment of the etching bath, which means the HF concentration is kept near to the initial concentration of 19.5 mol L^−1^ by means of adding the missing HF (evaporated or bound as H_2_SiF_6_). This procedure is necessary for keeping the etching parameters constant and, therefore, to allow homogeneous double layer formation.

The macroporous silicon etching bath contains HF with an initial concentration of 1.5 mol L^−1^ and acetic acid as a wetting agent. The F-ISE has been found here to generate precise results with no influence of the acetic acid. However, titration is not a suitable method for the HF determination in acetic acid containing etching baths. This is because the acetic acid is a buffering solution. Thus, addition of hydroxide ions (OH^-^) - as in the case of sodium hydroxide - or hydronium ions (H_3_O^+^) will not lead to a change in pH. Therefore, an adjustment of the pH in the required range is not possible, and no sufficient reaction of lanthanum nitrate with fluoride ions will occur. As a result, the HF content of acetic acid containing etching solutions can only be measured with F-ISE.

### Organic content

In electrochemical etching baths, hydrogen bubbles are formed on the surface of the silicon substrate during the process according to the following reaction [12]:

(2) $$\text{Si}+6\;\text{HF}+2\;{\text{h}}^{\text{+}}\to {\text{SiF}}_{6}^{}{}^{2-}\text{+}\;{\text{H}}_{2}\text{+}4\;{\text{H}}^{\text{+}}$$

For homogeneous pore formation, it is necessary to detach these hydrogen bubbles because, otherwise, they stick to the surface of the wafer and prohibit sufficient etching. The addition of surface wetting agents reduces the surface tension of the electrolyte, leading to a higher detachability of hydrogen bubbles [13]. Ethanol can serve as a surface wetting agent, as can acetic acid. A crucial factor for the evaluation of a suitable technique is the analysis method's capability of resistance against the aggressive HF.

The organic content of the baths is determined by the cuvette test method. This method can be applied for organic compounds in general, i.e., the method is capable of measuring organic as well as inorganic carbon. We focus on the organic carbon measurement due to the absence of inorganic carbon in our samples.

The method proves to be suitable for ethanol as well as for acetic acid solutions. HF does not disturb the measurements even at high concentrations, because the sample is highly diluted to conform to the measurement range. Despite the dilution, the cuvette test method is found to yield precise results with a relative error of 2.1 %. Disadvantages are the method's incapability of inline analysis and the required time of about 4 h for the analysis due to the temperature process.

**Figure 4:**
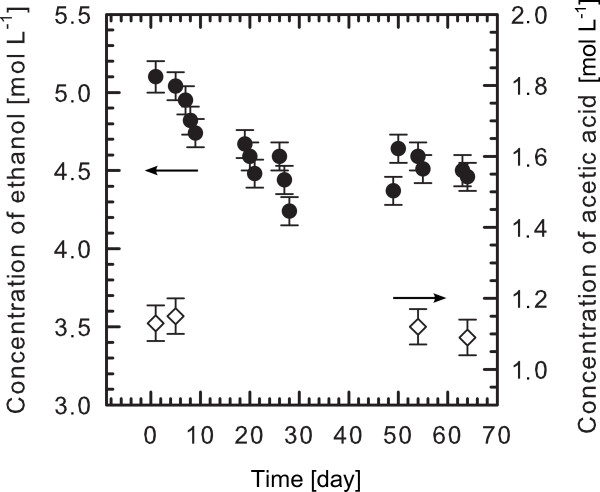
**Development of the organic contents in the etching baths for meso- and macroporous silicon.** Evaporation causes a decrease of the ethanol concentration in the mesoporous silicon etching bath of about 17 %; the step after 48 days is due to replenishment of the etching solution. The concentration of the acetic acid in the macroporous silicon etching bath is constant during the observation period due to a closed ‘bleed and feed’ reactor.

Figure 4 displays the development of the ethanol and the acetic acid concentration in the two investigated etching baths. A closed ‘bleed and feed’ reactor for macroporous silicon formation shows no decrease in acetic acid concentration over a period of 8 weeks. The ethanol concentration in the etching bath for mesoporous silicon was analyzed over a period of 8 weeks as well. However, we find a decrease of 17 % in ethanol concentration due to evaporation. The reactor for mesoporous silicon is not closed as HF and ethanol may form an explosive mixture. Hence, evaporation cannot be avoided completely. The step in the concentration of ethanol after 48 days in Figure 4 is due to replenishment of the etching solution.

## Conclusion

Achieving constant process conditions is crucial for the industrial production of porous silicon. For two commonly used etching bath compositions, i.e., high HF concentration in the presence of ethanol and low HF concentration in the presence of acetic acid, we determined suitable analysis methods. The HF content of etching solutions without acetic acid can be analyzed by titration or F-ISE, respectively; whereas, for acetic acid containing solutions, only F-ISE is useful due to unwanted reactions of the acetic acid with sodium hydroxide. The cuvette test method can be used for both surface wetting agents. The described methods withstand the aggressive HF and enable monitoring of the chemical composition of etching baths with adequate technical effort. This allows to keep the etching parameters for PSi double layers constant and to achieve homogeneous pore formation.

## Abbreviations

F-ISE, fluoride ion-selective electrode; H2SiF6, hexafluorosilicic acid; HF, hydrofluoric acid; PSi, porous silicon; TOC, total organic carbon.

## Competing interests

The authors declare that they have no competing interests.

## Authors’ contributions

JBN carried out the experiments, collected the data, and drafted the manuscript. SKS supervised the experiments. DWB and SKS supervised the entire work as the principal scientists and revised the manuscript critically. All authors interpreted the results together; they read and approved the final manuscript.

## Authors’ information

JBN is a PhD student at the ISFH; SKS is the head of the group for silicon thin film cells. DWB is a professor at the Institut für Technische Chemie at the Leibniz University of Hanover and head of the group for photocatalysis and nanotechnology.
